# Concrete constraints on abstract concepts—editorial

**DOI:** 10.1007/s00426-022-01685-9

**Published:** 2022-05-31

**Authors:** Anna M. Borghi, Samuel Shaki, Martin H. Fischer

**Affiliations:** 1 Department of Dynamic and Clinical Psychology, and Health Studies, Sapienza University of Rome, Via degli Apuli 1, 00185 Rome, Italy; 2 Institute of Cognitive Sciences and Technologies-CNR, Rome, Italy; 3 Ariel University, Ariel, Israel; 4 University of Potsdam, Potsdam, Germany

## Abstract

This special issue, "Concrete constraints of abstract concepts", addresses the role of concrete determinants, both external and internal to the human body, in acquisition, processing and use of abstract concepts while at the same time presenting to the readers an overview of methods used to assess their representation.

The contributions to this special issue, "Concrete constraints on abstract concepts," focus on concepts like "fantasy,” "duty," and "love." Although not dichotomously opposed to concrete concepts, abstract concepts are typically more detached from the senses, more variable, and their members are more heterogeneous. The topic of abstract concepts is fascinating both because of their prominent role in our cognitive activity and because they are currently thought to challenge embodied and grounded cognition views. Although we think that this challenge might be overcome, some cognitive domains, like language, emotions, and social interaction, might be more crucial for the representation of abstract than concrete concepts.

The novel contribution of this special issue to the current debate is that it addresses the role of concrete determinants, both external and internal to the human body, in processing and use of abstract concepts while at the same time presenting to the readers an overview of methods used to assess the cognitive representation of abstract concepts. Consistent with these goals, the issue is organized into three subsections—one on external influences, one on inner influences, and the last one on methodological novelties. This set of papers is preceded by an integrative review, the structure of which matches that of the special issue. Each subsection includes both theoretical and empirical articles. For the theoretical contributions, we have encouraged collaborations between authors from different labs, as well as cross-referencing between “schools”, to support a balanced presentation of different views. We outline below the structure of the special issue and briefly summarize the various contributions.

## An integrative review

The special topic is preceded by a review paper co-authored by Borghi et al. (under review), on concrete determinants of abstract concepts. After defining what they intend with abstract concepts and clarifying an analytically useful distinction between grounded, embodied, and situated cognition, the authors focus on the distinct roles that perception, action, language, and social interaction play in our cognitive competence with abstract concepts. The review offers a background to the entire special issue. Like the special issue, it addresses both external influences—the role of perception, action, culture, and social interaction—and internal influences—the role of interoception, metacognition, and inner speech—on abstract concepts processing and use. Finally, it highlights some selected methodological issues, focusing on promising interactive methods that can innovate the field and the importance of time course measures.

## Section 1: external influences

The first section addresses the following questions: which roles play perception, action, and sociality in acquiring and representing abstract concepts? What is the evidence for constraints from metaphors and expertise on abstract conceptual knowledge? The first theoretical contribution by Fischer et al. (2021) and Glenberg (2021) presents a paper and an accompanying demonstration video available at https://www.youtube.com/watch?v=C181DepjQf4&feature=youtu.be) on the teaching of numerical knowledge. An interesting and original new format characterizes this multi-medial contribution; the authors discuss a video in which a speaker, Art Glenberg (2021), illustrates to his students an abstract mathematical concept, regression to the mean, using a strategy inspired by embodied cognition. The authors aim to address whether a view based on simulation can account for phenomena concerning the abstract domain of mathematics. The video is taken as a proof-of-concept that shows how an embodied approach can be effective for teaching. Examples of adopted strategies are: referring to personal experiences, connecting with already familiar concepts, and frequently using gestures. The authors frame the discussion in the context of the distinction between grounded, embodied, and situated cognition. They provide a short review of developmental approaches to mathematical cognition and discuss how an embodied approach can represent a powerful tool for learning complex abstract notions.

In the second theoretical contribution, Troyer and McRae (2021) provide a broad overview focusing on the semantic relations associated with concrete and abstract concepts. Adopting the view according to which words are cues to meaning, they review the literature on written and spoken paradigms, showing that abstract concepts elicit multiple relations, that within these relations, social relations among people and relations within the self are particularly crucial, and that abstract concepts are quite heterogeneous. The authors argue that the field can move forward by analyzing different kinds of concrete and abstract concepts not in isolation but in relation to situated contexts, to real-time interaction, and by taking into account individual differences.

The theoretical contribution by Desai (2021) addresses the question of whether metaphors are embodied. The author offers a comprehensive review of research on the neural bases of metaphors, summarizing both neuroimaging and lesion studies. Next, he analyzed the grounding of metaphors in a variety of sensorimotor domains, including action, motion, texture, taste, and time. Desai concludes by arguing that metaphors are embodied. A different case is represented by idioms, the embodiment of which is more debatable and that, according to the author, are characterized by a different level of grounding.

The empirical contribution by Villani et al. (2021) examines cognitive differences between types of abstract institutional concepts. Both law experts and nonexperts rated several types of concepts: theoretical, institutional, food-related, and artifacts on several semantic dimensions. Not surprisingly, ratings of institutional concepts varied between law experts and nonexperts, with institutional concepts being perceived as more concrete and related to their sensorimotor experiences by the legal experts. More intriguing, however, is that pure-institutional concepts (e.g., parliament) showed higher ratings on sensorimotor experience while meta-institutional concepts (e.g., validity) showed a dominance of metacognitive and inner experience. The authors integrate their results into the current debate on the grounding of abstract concepts in sensorimotor or internal experience and also address broader theories of conceptual representations.

## Section 2: internal influences

The second section of this special issue addresses the question how internal processes such as inner speech, metacognition, and internal bodily signals can influence the acquisition and retrieval of abstract knowledge. The first theoretical paper by Dove, Barca, Tummolini, and Borghi focuses on the role of language in abstract concepts representation (Dove et al. 2020). Specifically, the article discusses two current theories, the WAT (Words As social Tools) and LENS (Language is an Embodied Neuroenhancement and Scaffold), as well as their claims, supporting evidence, and limitations. Both theories highlight the importance of both inner speech and overt language for abstract concepts and conceive language as a system that enhances cognition and helps the flexibility of our thought.

In the second theoretical contribution to this section, Monti et al. (2021) wrote a timely review on how signals from inside our bodies help establish our sense of self. They highlight the recently growing interest in interoceptive signal processing and review influential paradigmatic studies. This review inspires a distinction between material, social and spiritual self. Their focus on bodily constraints on self-representation not only informs competing theories of embodied cognition but also offers a new understanding of certain clinical conditions such as eating disorders and depersonalisation.

The empirical contribution to this section is co-authored by Muraki et al. (2020), who introduce the assumption that abstract concepts are not a unitary whole. To the best of our knowledge, they perform one of the first studies in which kinds of abstract verbs are considered (see also Villani et al., this issue). They use mental state, emotional state, and non-embodied state verbs and employ a syntactic classification and a memory task. Mental state verbs were both processed quickly in the syntactic classification task and memorized worse in the memory task. A further semantic richness analysis reveals interesting relationships between the concreteness of the associated words, their age of acquisition, and response times. Overall results are in keeping with multiple representation views that assign a crucial role both to grounded and linguistic dimensions.

## Section 3: methodological issues

What is the time course of cortical constraints on abstract knowledge retrieval? How can computational models inform cognitive theories on abstract concepts? The last section of the present special issue addresses these methodological questions. Three empirical papers focus on robotics and computational modelling and address either how abstract concepts are acquired or how they are represented. One further paper investigates the time course of access to sensorimotor information during abstract concepts processing with EEG.

The study by Pecyna et al. (2020) represents a developmental robotics approach in which the emergence of counting abilities is investigated as a result of different training conditions. Specifically, the authors investigated the contribution of pointing gestures and compared their findings against a previously published benchmark study with 4-year-old children. The humanoid iCub robot is used as a platform and shows that both sensorimotor signals and hand images as additional learning dimensions support the learning experience. The absence of differences between model data and child data is taken to confirm the plausibility of their embodied learning algorithm.

Next, Günther et al. (2020) describe a computational model for concrete and abstract concept representation, which combines systematic relations between semantic language representations and visual experience. In a series of three experiments, the authors present concrete and abstract words accompanied by two images: a model-selected image and a random image. Participants had to decide which image better represents the word's meaning. Participants' image preferences were in line with the model predictions for concrete and the most abstract words. According to the authors, the findings support a notion of grounding abstract words in which our previous visual experience can be extrapolated to the visual representation of abstract concepts.

A deep neural network model of the cortical areas crucial for sensorimotor, linguistic, and conceptual processing is presented by Henningsen-Schomers and Pulvermüller (2021). They model concrete concepts as more compact categories, endowed with many overlapping features, and abstract concepts as more heterogeneous, with only pairs of concepts sharing feature neurons. They found that, after learning, the central network, as compared to the peripheral ones, showed many shared neurons for concrete concepts; the opposite pattern, with central areas exhibiting relatively fewer neurons shared between pairs of category members, characterized abstract ones. Results have many implications for category learning as they might contribute to accounting for the difficulty of acquisition of abstract concepts.

In a final fine contribution to this special issue, Harpaintner et al. (2020) used event-related potentials (ERPs) to explore whether sensorimotor activity induced by abstract concepts reflects early access to conceptual information or later conceptual processes. In two experiments, implicit lexical decision and explicit conceptual decision tasks with abstract words were used to determine the degree to which recruitment of sensorimotor activity is task-dependent and flexible. The findings shed light on the time course of sensorimotor effects during abstract word processing. Specifically, early and late feature-specific ERP effects found with different onsets for both tasks supported the predictions of grounded cognition theories.
